# Potential of IMU Sensors in Performance Analysis of Professional Alpine Skiers

**DOI:** 10.3390/s16040463

**Published:** 2016-04-01

**Authors:** Gwangjae Yu, Young Jae Jang, Jinhyeok Kim, Jin Hae Kim, Hye Young Kim, Kitae Kim, Siddhartha Bikram Panday

**Affiliations:** 1 Industrial Engineering & Management Research Institute, Korea Advanced Institute of Science and Technology (KAIST), Daejeon 34141, Korea; ygjgy@kaist.ac.kr; 2 Department of Industrial & Systems Engineering, Korea Advanced Institute of Science and Technology (KAIST), Daejeon 34141, Korea; mbnmbckbs@kaist.ac.kr; 3 Department of Physical Education, Korea National Sport University, Seoul 05541, Korea; kimjhski@knsu.ac.kr; 4 Division of Liberal Arts and Science, Korea National Sport University, Seoul 05541, Korea; hykim@knsu.ac.kr; 5 Department of Physical Education, Seoul National University, Seoul 08826, Korea; dkrlcla9@snu.ac.kr (K.K.); ccdartha@snu.ac.kr (S.B.P.)

**Keywords:** IMU sensor, performance evaluation, stability, alpine skiing

## Abstract

In this paper, we present an analysis to identify a sensor location for an inertial measurement unit (IMU) on the body of a skier and propose the best location to capture turn motions for training. We also validate the manner in which the data from the IMU sensor on the proposed location can characterize ski turns and performance with a series of statistical analyses, including a comparison with data collected from foot pressure sensors. The goal of the study is to logically identify the ideal location on the skier’s body to attach the IMU sensor and the best use of the data collected for the skier. The statistical analyses and the hierarchical clustering method indicate that the pelvis is the best location for attachment of an IMU, and numerical validation shows that the data collected from this location can effectively estimate the performance and characteristics of the skier. Moreover, placement of the sensor at this location does not distract the skier’s motion, and the sensor can be easily attached and detached. The findings of this study can be used for the development of a wearable device for the routine training of professional skiers.

## 1. Introduction

The sports organizations of today are embracing their roles as entertainment providers [1,2]. Technology, in various forms, has been utilized in sports for many years and plays a particularly vital role in elite sports. Technological advancements at the elite level may flow down to consumers and also be used by the entertainment industry [3]. There are new types of gadgets to calculate the speed of a pitch, the strength of a putt, the arch of a basketball toss and the quality of a serve. These pro-level gadgets are coming to casual sports and eventually will create a new approach to improve performance, as well as a new way to entertain in sports [4].

Alpine skiing is one of the most popular sports at the Winter Olympic Games and is highly competitive; a difference of one hundredth of a second can change the standings. Alpine skiing is generally characterized by the repetition of ski turns, the basic motion for managing speed and direction. Consequently, the performance of turns greatly affects overall performance or a racer’s time; as a result, significant interest has been shown in the use of motion analysis of ski turns to improve performance. The biomechanical aspects of ski turns have been studied to understand the fundamental nature of the turn motion with several devices and methods [5,6]. In this study, we propose a method based on an inertial measurement unit (IMU) sensor for the analysis of turn performance that can be conveniently and effectively used by professional skiers while training. Specifically, this paper defines a professional skier as one who routinely competes in alpine ski competitions certified by the FÉDÉRATION INTERNATIONALE DE SKI(FIS).

The proposed study is motivated by the needs of professional skiers in the era of big data. It is well known that the German national soccer team analyzed the data collected during training sessions and identified individual players’ characteristics and performance. This data-oriented training and team operation was credited with the team’s victory in the 2014 World Cup [7]. With advancements in communication, sensors and wearable computing technologies, strong interest has been shown in the sports world to capture data while training and to use them to improve performance, prevent injuries and design strategies [8]. To date, ski motion analyses and data collection using sensors or digital devices have been studied only in controlled laboratory settings. Unlike a one-time experiment, however, data collection during daily training requires that the device should not distract from training, and acquisition and analysis must be done in a timely manner to provide rapid feedback to the skier. In this regard, the choice of which data to collect and how to best use the data while meeting these requirements is critical. This study is the first step in the development of an on-time feedback system for the use of professional skiers during their daily training.

Optical video-based motion capture has been widely used to analyze body motion in sports. For three-dimensional (3D) motion analysis, a marker-based motion capture system also has been used. These systems are effective for indoor experiments, but the use of such systems for sports performed in a wide outdoor area with high-speed movements, such as alpine skiing, has some limitations, because they require multiple cameras and light source settings.

Foot pressure sensors are effective tools that are widely used in ski experiments [9]. However, they have limitations in capturing motion data during skiers’ daily training. Attachment of pressure sensors to the boots or bindings may require special equipment or custom boots or bindings. The insole pad-type sensors also have some issues. Most professional alpine skiers use boots with a thermo-molded liner, or memory foam, that is precisely shaped for the individual skier’s foot for the best control of ski during the race. This liner perfectly fits the skier’s foot and, therefore, leaves no space for any sensors or device inside the boots. Moreover, even if a thin pressure pad can be placed inside the boots, skiers would be unable to replicate their best performance, because it would still make the skiers feel uncomfortable. As a result, pressure sensors may be used for experiments, but they may not be the best solution for everyday routine training.

**Figure 1:**
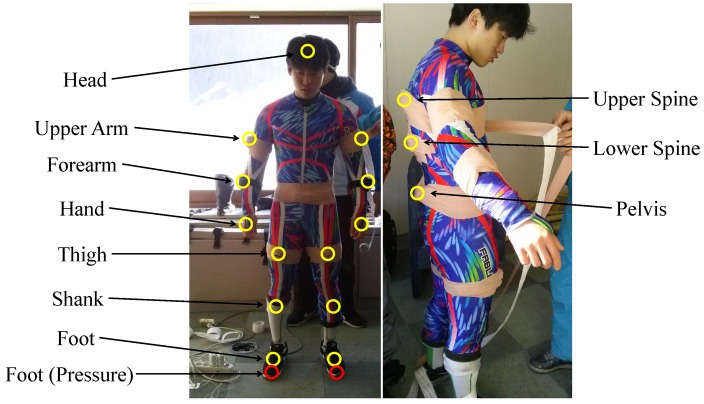
Sensors attached for full-body measurement.

Recently, IMU sensors, which measure acceleration and angular rotation, have gained attention for motion analysis in alpine skiing. In this paper, we first present the analysis to identify the best location to attach the IMU sensor to the skier’s body. The best location is defined as the location at which the sensor can collect data for the analysis of ski turns, while not interrupting the skiers’ performance. It must also be easy to attach and detach the sensor for convenience in the skiers’ everyday training. To logically identify the optimal IMU location, we conducted experiments with a professional skier who had experience in the final rounds of FIS-certified competitions. Multiple sets of data were collected from 16 IMU sensors attached to the skier, as shown in Figure 1, while he conducted typical on-slope training on an actual alpine race track in Pyeongchang Resort, where the 2018 Winter Olympics will take place. Extensive statistical analyses were performed to identify the best sensor location. We then show that the data from the proposed location on the body characterize the ski turn and its performance. To the best knowledge of the authors, this is the first report on performance analysis with data collected from IMU sensors attached in a full-body configuration on a professional skier at an actual racing track.

In summary, we seek to answer the following questions:

1. What is the best location on the body to attach an IMU sensor to capture the key characteristic of the turns in alpine skiing?
2. From the selected location, how should data be analyzed to evaluate the skier’s performance, particularly the lateral-asymmetric test and the adaptation effect of training?

The lateral-asymmetry is defined as the discrepancy between left and right turns, and it is often considered to be a characteristic that affects the performance of ski turns [10]. The adaptation effect refers to performance improvement that occurs with more trials as skiers become accustomed to slope conditions, such as snow quality.

The contribution of this paper is clear. It presents a method for the effective use of an IMU sensor to analyze the performance of professional skiers. In addition, the optimal sensor location and methods of analysis open new opportunities for the development of a personal sporting device using a sensor and smart phone, such as a training device based on the Internet of Things. This further development and application are discussed in the last section.

The paper is organized as follows. Section 2 summarizes the research on the motion analysis of alpine skiing using various devices. Section 3 describes the experimental procedures and methods. Section 4 presents the analysis of the sensor data to identify the best location on the body to attach an IMU sensor. Section 5 presents the validation of the proposed location by proving statistically that it can capture the characteristics. Finally, Section 6 summarizes the study and discusses the potential uses of IMU sensors in the ski training of professional alpine skiers.

## 2. Literature

### 2.1. Motion Analysis Using Different Experimental Devices

Traditionally, biomechanical analysis of motion in alpine skiing has been conducted with video cameras and force (pressure) sensors. One of the early studies in the biomechanical analysis of ski turns was conducted by Maxwell and Hull [11], who used transducers to estimate the strength and loading on the knee during the ski turns. Schaff and Hauser [12] used video-based analysis to discover significant differences in the biomechanical aspects of the knee joint by turn techniques. Similarly, Müller *et al*. [13] used video-based analysis to reveal statistical differences in the biomechanical aspects of turn techniques between experienced and intermediate skiers. Yoneyama *et al*. [14] used a specially-designed system with angle sensors and force plates to compare the differences in joint motion and reacting forces in the legs between carving and conventional turns. Müller and Schwameder [5] used video cameras and force plates to discuss the joint motion of body parts and the action-reaction forces on a skier’s body when performing carving turns. Scott *et al*. [15] used pressure sensors to measure the action of forces between the ski plates and the snow surface. Vaverka and Vodickova [10] used pressure sensors to measure the forces that act on the legs to discuss the effects of laterality on performance. For 3D motion analysis, reflective markers are used in addition to the video-based analysis. Klous *et al*. [6] used a marker-based motion capture system and force plates to analyze in 3D space the load placed on the knee by different turn techniques. Park *et al*. [16] adopted a marker-based motion capture system to analyze the biomechanical aspects of the legs in 3D space during ski turns.

Recently, IMU sensors have gained attention for their potential use in motion analysis of ski turns. Brodie *et al*. [17] used IMU sensors to analyze the biomechanical aspects of the ski turns. Krüger and Edelmann-Nusser [18] studied the accuracy of a full-body inertial measurement system in measuring the motions during alpine skiing in comparison with that of an optical video-based system. Kondo *et al*. [19] used IMU sensors to calculate skiers’ joint angles during two different turn techniques.

However, previous research using IMU sensors has intended to analyze ski turns and motions in one-time only experimental environments. Although those methodologies have allowed for robust technique analyses, the number of sensor units and total weight of the equipment used appears to be prohibitive for use in daily training environments or during competitions. These issues have been addressed in previous studies, such as [20,21]. Unlike the preceding research, the goal of our study is to identify the best IMU sensor location. We also conduct turn and motion analyses, but as part of the process of identifying an optimal location.

The work of Marsland *et al*. [20] is the most similar to this study. Their goal was to determine whether a single micro-sensor unit attached to the body could be used to effectively identify each of the main techniques used during a cross-country skiing competition. Although they studied cross-country skiing and we study alpine skiing, they also tried to identify the best sensor location. However, they did not perform a comparison analysis of possible sensor locations. Instead, they selected a single location, the upper back, and argued that it was acceptable by showing that it allowed the sensor to identify the intended motions of the cross-country skier.

### 2.2. Performance Analysis Using Different Experimental Devices

A few studies have focused on the performance of turn techniques. Michahelles and Schiele [22] developed a system called *SKI* to measure the edge angles, velocities and foot pressures to determine the center of mass. This system of different types of sensors was designed to effectively provide information on the strengths and weaknesses of a skier. Kirby [23] developed a system called *vLink* to measure the acceleration and the angle between the moving direction of the body and the skis for real-time feedback. Supej [24] developed a system with an IMU sensor to retrieve a reference trajectory of the skier’s path. Federolf [25] suggested an analytical method based on video analysis with which to quantify the performance of skiers based on the speed and distance traveled. Debski [26] introduced a simulation-based trajectory optimization algorithm to suggest the optimal path for better performance in ski races. Nemec *et al*. [21] used a machine learning technique to develop reference postures that can be adopted in coaching and training in alpine skiing.

In summary, previous studies of alpine skiing with measurement devices have mainly focused on understanding the biomechanical aspects of turn motions. They have attempted to provide insight into the effects of body motion and posture on performance. In addition, some studies have focused more on the performance side of ski turns, and although IMU-based measurements have gained attention in academic research and commercial applications, no study has logically identified the optimal IMU location for ski training.

The ultimate goal of this study is to offer a foundation for the development of a sensor-based feedback analyzer that can be used by professional skiers during their daily training.

## 3. Description of the Experiment

To identify the best location on the body of a skier to place an IMU sensor, we designed an experiment in which a professional skier wearing IMU sensors performed a set of downhill turns as a part of routine on-slope training. Note that the insole type of foot pressure sensor is not appropriate for use in the everyday training of professional skiers, as discussed in the previous section. However, in our experiments, we attached the foot pressure sensors inside the special boots and collected weight shift data from them. Because the skier’s weight shift and turn characteristics can be well identified from a pressure sensor on the foot, we used the data collected from the foot pressure sensor as the reference to compare to the data from the IMU sensors to identify the best IMU location on the body. We confirmed that although the skier did not want to use the foot pressure sensors to collect data during daily training, due to the time required for wearing the specially-designed boots, he confirmed in the post-survey that they did not influence his performance during the experiment.

The participant was a 20-year-old male professional skier with a height of 175 cm and a weight of 73 kg, who had experience competing in an FIS-certified competition and was a member of the alpine ski team at the Korea National Sports University (KNSU) [27]. Before the measurements began, the participant was given a full explanation of the research purpose and experimental procedure and signed Institutional Review Board consent forms to comply with the ethical principles of the Declaration of Helsinki (1975, revised 1983).

**Figure 2:**
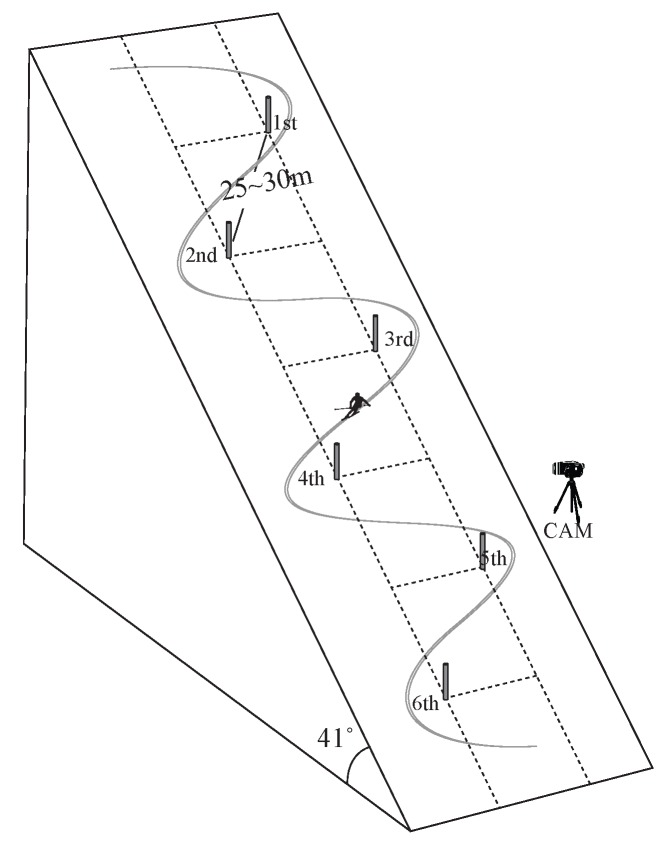
Graphical representation of the experimental setup.

The experiment was conducted on the Rainbow 3 slope at Yongpyeong Resort, Pyeongchang, Gangwon-do, Korea. The official average slope angle is $41^{\circ }$. Six poles were installed to simulate the giant slalom, which has straight distances between 25 and 30 m. In our experiment, we attempted to install the poles with nearly the same widths and lengths to provide the same environment for left and right turns, which would be a sufficient condition for determination of the laterality. Figure 2 is a graphical representation of the experimental setup described above. All of the experiment trials were recorded with a video camera, as indicated in Figure 2, to validate the turn motions.

We used a set of 16 IMU sensors (myoMOTION, Noraxon, Scottsdale, USA) for full-body measurement. Each sensor can measure linear and angular accelerations in 3D space. The sampling rate used in our experiment was 100 Hz. For the foot pressure sensor, we used an insole-type sensor system from Moticon that has 13 distinct sensors. Each sensor measures the force applied in N/cm${}^{2}$. The sampling rate used in our experiment was 50 Hz. A summary of the specifications is given in Table 1.

The myoMOTION, a 3D multi-joint motion analysis system using IMU sensors, is one of the most widely-used IMU sensor systems in academic research. Recent academic work performed with the system is described in [28]. In the post-processing, to calculate the rotational angles and positions, the values from the accelerometer, magnetometer and gyroscope must be combined. The data from the accelerometer and magnetometer are used to determine the initial position, and the gyroscope’s angular acceleration value is integrated to calculate the angle. To correct the drift error that might occur during the integration process, a Kalman filter is applied. A more detailed description of the method can be found in the technical report and specification produced by Noraxon [29,30].

With this setting, the skier performed six turns: three right turns and three left turns. For each trial, the sensors taped to the skier were tightly secured again to make sure that they were attached to the body. Then, the position was reset in the proper standing position. Note that the relative rotation angles and positions were measured from this reset position. An additional turn (to the right) was performed after the turns without a pole to slow down. This last turn is not included in the analysis. In summary, a total of six experimental turns for each trial and a total of nine trials were attempted; the second trial was excluded because we discovered errors in the data collection. As a result, eight trials of six turns each are used for the analyses. Once the experiments were performed, the data were collected from the sensors, and post-processing was conducted offline.

## 4. IMU Location Analysis

**Figure 3:**
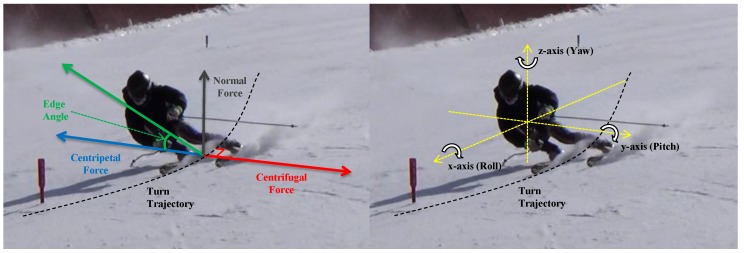
Physics and the rotational axes of the ski turn.

Typically, the motion of a ski turn can be decomposed into 3D rotations, as described in Figure 3.

Among these, the rotation over the x-axis or the roll is considered to be the key motion that affects the performance of ski turns. This can be easily understood by considering the physics of a ski turn as the roll generates the centripetal force to resist the centrifugal force that drags the skier out of the circular path of the ski turn. Consequently, maintaining stable progress by keeping a balance between the two forces allows postural stability and the performance of ski turns. The significance of the roll angle and the importance of the roll motion in ski turns are well supported by previous studies [31,32].

The following analyses were preformed to identify the best IMU location to identify the roll angle. Note that these analyses are complementary and do not replace one another.

1. Pattern correlation analysis;
2. Clustering analysis;
3. Turn detection analysis.

The pattern correlation and cluster analyses were performed to investigate the similarity between the patterns from the IMU sensors and those from the foot pressure sensors. The turn detection analysis identifies the IMU location at which the turn patterns are best recognized.

### 4.1. Pattern Correlation Analysis

**Figure 4:**
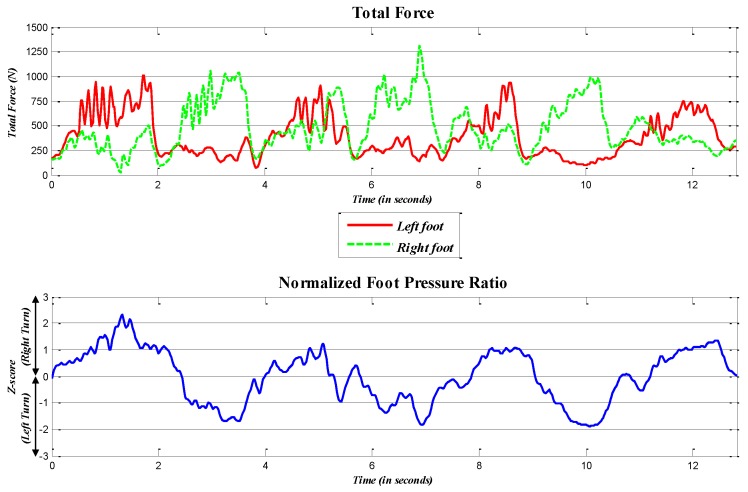
Total foot forces of Trial 8 and the normalized foot pressure ratio.

As mentioned in the Introduction, numerous studies support the effectiveness of foot pressure sensors in capturing a skier’s turn pattern, especially the roll motion [5,6,10,12,14,15,22]. Therefore, in this analysis, the roll angle data collected from each IMU sensor are compared to those from the foot sensors. The upper panel in Figure 4 shows the foot pressure values collected from the foot pressure sensors. As we are concerned with the pattern of weight shift in the roll motion, the values from left and right turns are combined to evaluate the pressure ratio. We denote ${\hat{F}}^{t}$ as the set of the foot pressure ratio for trial *t*, consisting of ${\hat{f}}^{t}\left(n\right)$, which is the *n*-th time index, such that:

(1) $${\hat{F}}^{t}=\left\{{\hat{f}}^{t}\left(1\right),{\hat{f}}^{t}\left(2\right),...,{\hat{f}}^{t}\left(N^{t}\right)\right\}$$

where $N^{t}$ is the total number of data points in trial *t*. Likewise, the data from the IMU sensor at location *l* in trial *t* are denoted as ${\hat{M}}_{l}^{t}$, and each data point is represented as ${\hat{m}}_{l}^{t}\left(n\right)$, such that:

(2) $${\hat{M}}_{l}^{t}=\left\{{\hat{m}}_{l}^{t}\left(1\right),{\hat{m}}_{l}^{t}\left(2\right),...,{\hat{m}}_{l}^{t}\left(N^{t}\right)\right\}$$

These datasets are smoothed with a five-point moving average to reduce the data noise, which is a frequently-used method of signal processing in alpine skiing studies [18,33,34].

**Figure 5:**
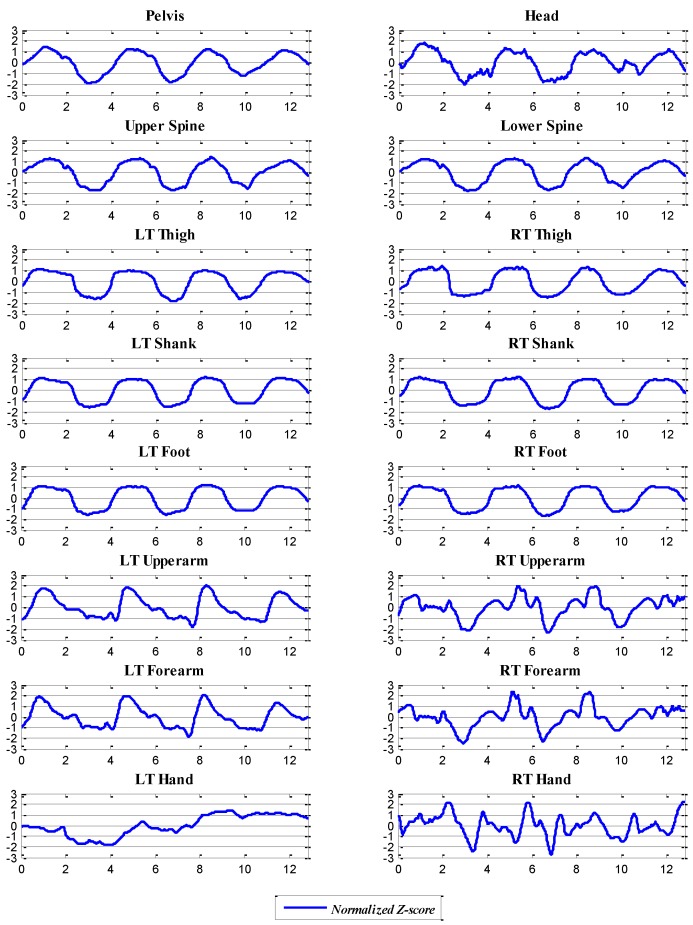
Example of the normalized IMU data (Trial 8) for the roll motion; the x-axis is the time, and the y-axis indicates the normalized roll angle, $M_{l}^{t}$.

To compare the roll motion patterns among the different sensors, we normalize the dataset using the standard score normalization method, and the following equation is used:

(3) $$Z=\frac{x-\hat{\mu }}{\hat{\sigma }}$$

where *Z* is the normalized score of each data point *x* in a dataset and $\hat{\mu }$ and $\hat{\sigma }$ represent the average and standard deviation of the dataset, respectively. We use the standard score method to normalize the data, because it is robust to the outliers and extreme values frequently detected in measurements of dynamic motions, such as those involved in alpine skiing [35]. This method is also common in the sports research literature, such as in [36,37]. The normalized datasets of the foot pressure ratio and IMU data are represented as $F^{t}$ and $M_{l}^{t}$, respectively. The normalized foot pressure ratio is shown in the lower panel of Figure 4, and the normalized IMU data at each location are shown in Figure 5.

The correlation value, denoted by $\rho_{l}^{t}=\rho \left(F^{t},M_{l}^{t}\right)$, is then evaluated between the normalized foot pressure ratio, $F^{t}$, and each normalized IMU data, $M_{l}^{t}$ at trial *t*, such that:

(4) $$\rho_{l}^{t}=\rho \left(F^{t},M_{l}^{t}\right)=\frac{\sum_{n=1}^{N^{t}}\left(\frac{f^{t}\left(n\right)-{\hat{\mu }}_{F}^{t}}{{\hat{\sigma }}_{F}^{t}}\right)\left(\frac{m_{l}^{t}\left(n\right)-{\hat{\mu }}_{M_{l}}^{t}}{{\hat{\sigma }}_{M_{l}}^{t}}\right)}{N^{t}-1}$$

where ${\hat{\mu }}_{F}^{t}$ is the sample mean and ${\hat{\sigma }}_{F}^{t}$ is the sample standard deviation of the dataset $F^{t}$. Similarly, ${\hat{\mu }}_{M_{l}}^{t}$ is the sample mean and ${\hat{\sigma }}_{M_{l}}^{t}$ is the sample standard deviation of the dataset $M_{l}^{t}$.

Finally, we calculated the correlation between the normalized foot pressure ratio and the normalized IMU data by Equation (4). Table 2 shows the resultant correlation values for each body location for each trial. As shown in Table 2, the sensors located on the spine and lower body (spine, thigh, shank, foot and pelvis) have high correlation values. That is, the roll motion measured at these parts of the body and the weight shift, which is the key motion of the ski turns, are closely related.

### 4.2. Clustering Analysis

We also performed a hierarchical clustering analysis. In the previous correlation analysis, the turn pattern from each IMU sensor was compared to that from the foot pressure sensors. However, in the hierarchical clustering analysis, the turn patterns among the IMU sensors and the foot pressure sensors were compared to one another, and the similarities were measured. One of the goals of this hierarchical clustering analysis was to validate the pattern data by analyzing the similarity measures. In the analysis, for the similarity distance, we used *correlation distance*, which is often used in hierarchical clustering when the correlations among the objects are known. To cluster the body locations, we specifically considered the median correlation value of each body location to reduce the effects of outliers, given the small sample size. This median correlation value is denoted as $R_{l}$ for each body location *l*, where *R* is between −1 and one. The definition of the correlation distance in the hierarchical clustering is $1-R_{l}$, and its range is thus between zero and two. For the clustering method, we used centroid values to measure the distances between the clusters based on Equation (5).

(5) $$d\left(r,s\right)=|{\bar{x}}_{r}-{\bar{x}}_{s}|,\;\;\;\mathrm{where}\;\;\;{\bar{x}}_{r}=\frac{\sum_{n=1}^{N_{r}}x_{r}}{N_{r}}$$

In this equation, d(r,s) indicates the distance between cluster *r* and *s*, where ${\bar{x}}_{r}$ is the centroid of cluster *r* with $N_{r}$ objects.

**Figure 6:**
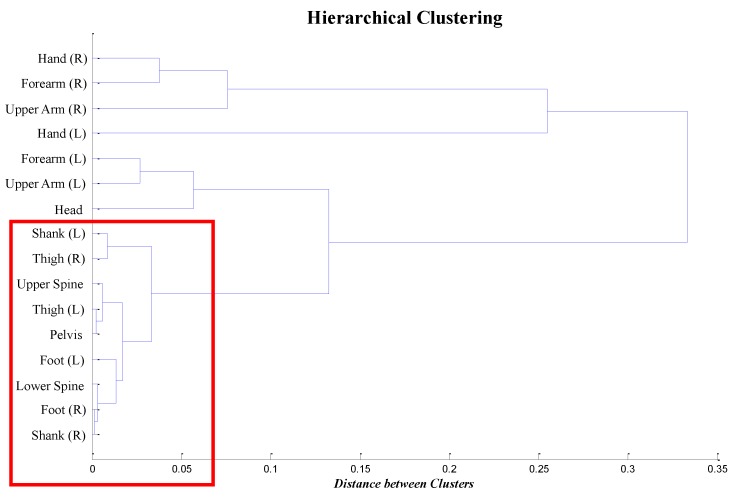
Hierarchical clustering result of IMU sensors based on the correlation distance.

The result is described in Figure 6. From the result, it can be inferred that the IMU sensors located on the spine and legs and those of the foot pressure sensors (indicated in the red box in the figure) produced very similar data patterns. Note that the correlation values of these locations in Table 2 are over 0.80, which is conventionally acceptable for judging the existence of a strong correlation [38,39].

### 4.3. Turn Detection Analysis

**Figure 7:**
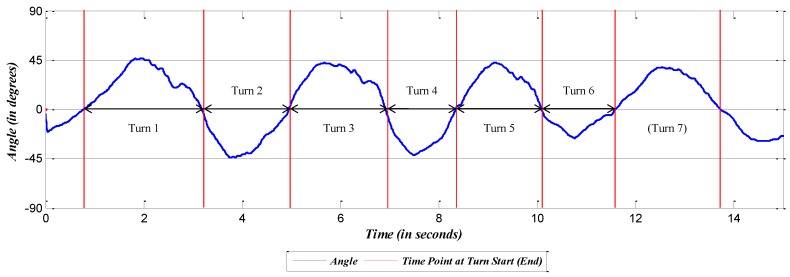
Plot of IMU data at the pelvis in Trial 8.

The previous analyses identified the IMU sensors on the spine and lower body as good candidates. In the final analysis, we tested how well these candidate sensors can recognize ski turns. We first define the term *turn-detection*, which compares the actual number of turns and the turns detected by the sensors. To measure this turn-detection, the *zero-crossing* method is used, which can be explained by the example in Figure 7. Figure 7 is the roll angle from the IMU on the pelvis in Trial 8. A negative value of the roll indicates that the skier was leaning to the left, and a positive value indicates that the skier was leaning to the right. Considering the data pattern of the plot, the turn can be defined as the interval between two zeros or the two zero-crossings.

As an example of the turn-detection measure, seven turns are recognized by counting the zero-crossings in the data of Figure 7, and the actual number of ski turns is seven. Therefore, in this case, we quantify that the turn is identified in all seven instances: 7/7 = 100%. If more or fewer turns are identified, the turn-detection measure will be more or less than 100%, respectively. The result of the turn-detection measurement for each sensor in each trial is listed in Table 3.

**Figure 8:**
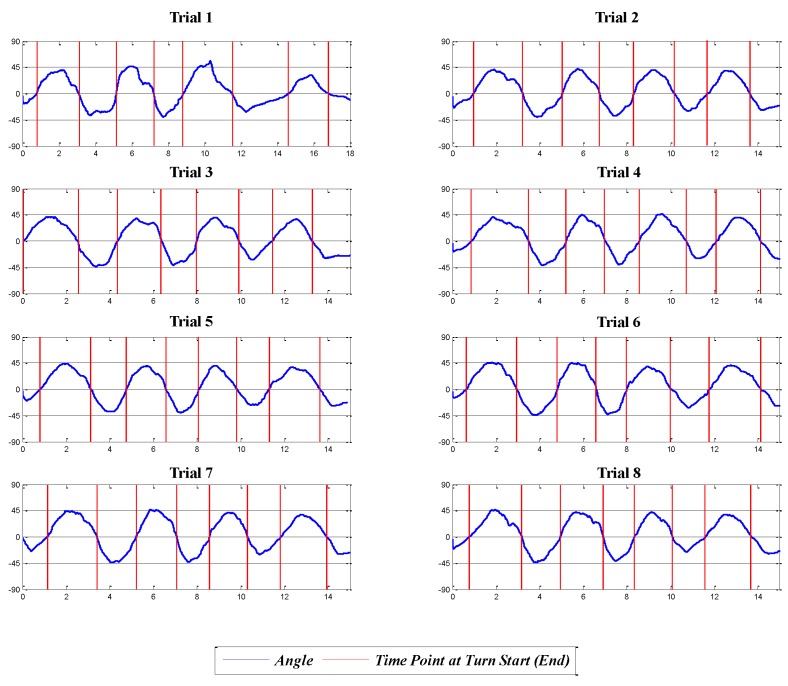
The roll angle pattern from the IMU at the pelvis: x- and y-axes represent the time and angle of the roll motion, respectively.

From this table, it can be seen that the pelvis, right shank and left and right feet are the good candidate locations that show 100% turn-detection. However, we select the pelvis as the best location for the IMU sensor, because it is the location at which the skier felt the least distraction. Furthermore, the feet or shanks require a pair of sensors on each side of the leg, which demands additional effort for attachment and detachment of the sensors and for analysis of the data. In addition, our survey indicates that the IMU sensors attached to the feet (the ski plates) distracted from the performance of ski turns and that the pelvis was considered to be the most convenient and least distracting location, as indicated in Table C1 in Appendix C. Figure 8 shows the data from the IMU sensor attached to the pelvis for the eight valid trials.

## 5. Skiing Performance Analysis and Validation

From the sensor location analysis, we identified that the pelvis is the best location on the body to attach an IMU sensor for the detection of turn characteristics regarding performance. The next question is “Can the proposed IMU sensor location capture the turn characteristics regarding the performance?” Answering this question could also validate that the pelvis is the best location for an IMU during skiers’ daily training. We conducted two performance analyses based on the data collected from the IMU sensor at the pelvis:

1. Lateral-asymmetric performance test;
2. Test for the adaptation effect of training.

### 5.1. Lateral-Asymmetric Performance Test

*Lateral-asymmetry*, which defines the discrepancy between left and right turns, is often considered to be a characteristic that affects the performance of ski turns [10]. Before the experiment, we first conducted an in-depth interview with the skier to determine his known turn characteristics. We then attempted to analyze whether the data captured from the pelvis can identify the characteristics. From the interview, the participant mentioned that he was less confident on right turns (where the outer leg is the left leg) than left turns due to the aftereffects of inveteratedisc surgery on the left lumbar as listed in Table C1 in Appendix C. Although we cannot determine the effects on left and right turns in an absolute numerical value, we can at least speculate that there might be a difference between left and right turns in terms of time and stability. Therefore, it is reasonable to hypothesize that there is a difference or lateral-asymmetry in the performance of left and right turns.

For the hypothesis tests based on the IMU sensor, the time duration of each turn is used for the performance measure. For the hypothesis test based on the foot pressure sensor, the maximum foot pressure ratio is used for the performance measure. The time duration of each turn and the maximum foot pressure ratio of each turn are summarized in Table 4.

With these data, two hypothesis tests are performed. The first hypothesis is that no time difference exists between left and right turns, such that:

(6) $$H_{0}:\mu_{L}^{*}=\mu_{R}^{*}\;\mathrm{vs}.\;H_{1}:\mu_{L}^{*}\neq \mu_{R}^{*}$$

That is, $\mu_{L}^{*}$ and $\mu_{R}^{*}$ are the true mean of the left and right time durations, respectively. The second hypothesis is that there is no difference in the foot pressure between left and right turns, such that:

(7) $$H_{0}:\mu_{L}^{+}=\mu_{R}^{+}\;\mathrm{vs}.\;H_{1}:\mu_{L}^{+}\neq \mu_{R}^{+}$$

where $\mu_{L}^{+}$ and $\mu_{R}^{+}$ are the true mean of the left and right maximum foot pressure ratio, respectively. Specifically, the foot pressure on the right foot should be higher in left turns, and *vice versa*, allowing us to capture the maximum foot pressure ratio of the right foot for each left turn and the maximum pressure ratio of the left foot for each right turn. Then, these maximum values are compared for the hypotheses testing.

Because we had three left turns and three right turns for each trial and a total of eight trials, a total of 24 left turns and 24 right turns were available. With this comparably small sample size, we planned to conduct two-sample *t*-tests. Kolmogorov–Smirnov tests (K-S tests) were conducted first to check the normality of the population data. A variance test was also conducted to check whether the population variances were equal for the accurate calculation of *t*-statistics. The detailed procedures are described in Appendix A. We proved the normality of the population data in Appendix A.1. Furthermore, we showed that the population variances in time duration data for left and right turns are equal, while the population variances of the foot pressure ratio for left and right turns are not equal in Appendix A.2.

We conducted two-sample *t*-tests with a significance level (*α*) of 0.05.

- Hypothesis test for the time duration:
  In the hypothesis test from Equation (6), the null hypothesis, $H_{0}$, is that there is no significant difference in the time duration between left and right turns $\left(\mu_{L}^{*}=\mu_{R}^{*}\right)$. The alternative, $H_{1}$, is that there is a significant difference in the time duration between left and right turns $\left(\mu_{L}^{*}\neq \mu_{R}^{*}\right)$. Because the population variances of the time duration data for left and right turns are equal, the test statistic, $T_{0}^{*}$, is defined as the following Equation (8). In this equation, ${{\hat{\mu }}_{L}}^{*}$ and ${{\hat{\mu }}_{R}}^{*}$ indicate the sample means of the time duration in left and right turns, whereas ${s_{L}^{*}}^{2}$ and ${s_{R}^{*}}^{2}$ indicate the sample variances of the time duration in left and right turns. $n_{L}^{*}$ and $n_{R}^{*}$ are the number of samples for each dataset.
  (8) $$T_{0}^{*}=\frac{{{\hat{\mu }}_{L}}^{*}-{{\hat{\mu }}_{R}}^{*}}{S_{p}\sqrt{\frac{1}{n_{L}^{*}}+\frac{1}{n_{R}^{*}}}}\;\mathrm{where}\;S_{p}=\sqrt{\frac{\left(n_{L}^{*}-1\right){s_{L}^{*}}^{2}+\left(n_{R}^{*}-1\right){s_{R}^{*}}^{2}}{n_{L}^{*}+n_{R}^{*}-2}}$$
  Finally, the calculated test statistic is 4.27, and its absolute value, $|T_{0}^{*}|$, is larger than $t_{0.025}\left(n_{L}^{*}+n_{R}^{*}-2=46\right)≅2.01$, such that it falls into the rejection region. Thus, the null hypothesis that there is a significant difference in the time duration between left and right turns is rejected.
- Hypothesis test for the maximum foot pressure ratio:
  Following the hypothesis test of Equation (7), the null hypothesis, $H_{0}$, is that there is no significant difference in the maximum foot pressure ratio between left and right turns $\left(\mu_{L}^{+}=\mu_{R}^{+}\right)$. The alternative, $H_{1}$, is that there is a significant difference in the maximum foot pressure ratio between left and right turns $\left(\mu_{L}^{+}\neq \mu_{R}^{+}\right)$. Because the population variances of the maximum foot pressure ratio data for left and right turns are not equal, the test statistic, $T_{0}^{+}$, is defined as Equation (9). In this equation, ${{\hat{\mu }}_{L}}^{+}$ and ${{\hat{\mu }}_{R}}^{+}$ indicate the sample means of the maximum foot pressure ratio in left and right turns, whereas ${s_{L}^{+}}^{2}$ and ${s_{R}^{+}}^{2}$ indicate the sample variances of the maximum foot pressure ratio in left and right turns. $n_{L}^{+}$ and $n_{R}^{+}$ are the number of samples for each dataset.
  (9) $$T_{0}^{+}=\frac{{{\hat{\mu }}_{L}}^{+}-{{\hat{\mu }}_{R}}^{+}}{\sqrt{\frac{{s_{L}^{+}}^{2}}{n_{L}^{+}}+\frac{{s_{R}^{+}}^{2}}{n_{R}^{+}}}},\mathrm{where}\;\mathrm{the}\;\mathrm{degree}\;\mathrm{of}\;\mathrm{freedom}\;v^{+}=\frac{{\left(\frac{{s_{L}^{+}}^{2}}{n_{L}^{+}}+\frac{{s_{R}^{+}}^{2}}{n_{R}^{+}}\right)}^{2}}{\frac{{\left(\frac{{s_{L}^{+}}^{2}}{n_{L}^{+}}\right)}^{2}}{n_{L}^{+}-1}+\frac{{\left(\frac{{s_{R}^{+}}^{2}}{n_{R}^{+}}\right)}^{2}}{n_{R}^{+}-1}}$$
  Finally, the calculated test statistic is 2.62, and its absolute value, $|T_{0}^{+}|$, is larger than $t_{0.025}\left(v^{+}=35\right)≅2.03$, such that it falls into the rejection region. Thus, the null hypothesis that there is a significant difference in the maximum foot pressure ratio or the balance in weight shifts between left and right turns is rejected.

A summary of the two hypothesis tests is presented in Table 5. With a significance level (*α*) of 0.05, both null hypotheses are rejected. That is, both tests indicate a difference between left and right turns.

In summary, the two hypothesis tests show a significant difference or lateral-asymmetry in the performance measures, the time duration and the maximum foot pressure ratio. Because the results are identical, it can be statistically inferred that the IMU sensor at the pelvis is as capable of analyzing the lateral asymmetric performance as the foot pressure sensors.

### 5.2. Test for the Adaptation Effect of Training

We also conducted an analysis to validate whether the IMU sensor can capture the adaptation effect of training on performance. For the analysis, we conducted another interview with the participant after the experiments regarding self-assessment of the performance of each trial, as listed in Table 6. In the self-assessment, we let the skier evaluate the performance of each trial on a scale of one to 10 (one is the worst, and 10 is the best). Notice that as indicated earlier, the data from the IMU during the second experimental attempt were not properly collected, and the second attempt is therefore indicated with an asterisk in the table. However, we included the self-assessment in the table and considered the fact that the skier made the attempt during the experiment in the analysis, because the adaptation effect depends on the number of trials. Specifically, although there is no record for the second attempt, we considered the second attempt to give a total of nine attempts. As indicated in Table 6, the skier mentioned in the interview that his performance seemed to improve as more trials were performed, because he grew accustomed to the experimental devices and the slope conditions, such as the snow quality, as the trials proceeded. Because the self-assessment is independent from the failure of our actual second experiment, we included the self-assessment score for the actual second trial (marked *).

**Figure 9:**
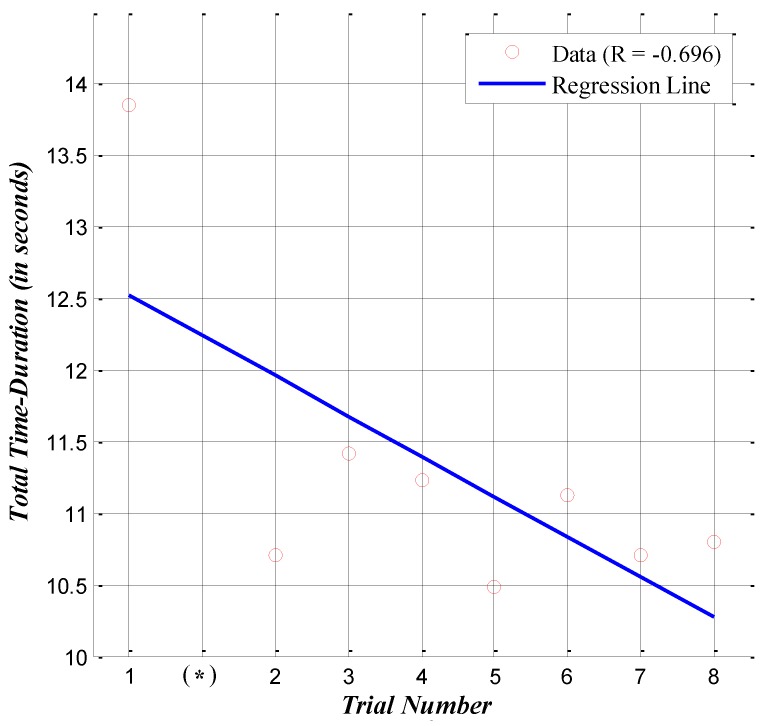
Plot of the correlation analysis between trial number and total time duration.

Based on this result, the following hypothesis is made: the skier adapted to the test environment, and his turn performance improved as the experiment proceeded. Figure 9 shows the time duration for each trial based on Table 4. Each dot indicates the time duration, and the line depicts the linear regression result. As mentioned above, in Figure 9, the second attempt marked with an asterisk on the x-axis does not have data, but we considered the fact that an attempt was made; therefore, there is a total of nine experimental trials. The correlation value is −0.696. This result indicates that the time duration data collected from the IMU at the pelvis captured the adaptation effect. The validation of the correlation analysis is explained in Appendix B.

## 6. Conclusions

In this paper, we first present an analysis in which the best location of an IMU sensor on the skier’s body was identified. The best location is defined as the body part at which the sensor can effectively collect the data for the analysis of a turn motion without interrupting the skier’s performance. It must also allow easy attachment and detachment of the sensor for the convenience of daily training.

In the series of analyses, we identified the pelvis as the best location to attach the IMU sensor, because the data collected from this location effectively address the performance measure and the characteristics of the skiers. The pelvis was also the preferred location of the skier, because it creates less of a distraction for the skier during a race.

Although the test was performed with only one participant, and we understand that more objective conclusions could be drawn from more participants, an experiment with a single participant still has value. Some studies of alpine skiing have been based on single-subject experiments, including [14,15,17,19], and analyzing an experiment with a single subject is not unusual in ski research. Another rationale for performing our experiment with a single subject is that the results can provide the direction for future multiple-subject experiments.

Moreover, in the era of smartphones and numerous new wearable devices, as described in [40], the findings of this study are significant. They can lay the ground work for the development of a new wearable device for ski training. The proposed analysis methods can be easily adopted, because they are not computationally demanding. Therefore, they can be used to design a system to give numerous types of feedback for skiers’ performance during training.

In summary, the value of our research comes from its use of a professional skier and its establishment of new possibilities for performance analysis in alpine skiing using IMU sensors. Because the effectiveness of IMU sensors in the analysis of ski turns is analytically proven, the effects of other signals of the IMU sensor, including angular/linear velocities and accelerations, on performance and the yaw motion analysis of ski turns can be interesting research topics. We reserve these topics for future study.

## Figures and Tables

**Table 1:** Specifications of the sensors used in the experiment.

**Specification (IMU)**	
Channel	Up to 18 sensors
Static accuracy	$\pm 0.4^{\circ }$
Dynamic accuracy	$\pm 1.2^{\circ }$
Sampling frequency	100 Hz
Data output	Joint angles, acceleration, quaternions
**Specification (Foot Pressure)**	
Channel	13 sensors per insole
Sensitivity	0.25 N/cm${}^{2}$
Coverage	Up to 50%
Range	0.0∼40.0 N/cm${}^{2}$
Sampling frequency	50 Hz
Principle	Capacitive

**Table 2:** Correlation values of roll motion by the sensor locations.

Category	Trial 1	Trial 2	Trial 3	Trial 4	Trial 5	Trial 6	Trial 7	Trial 8	Mean	Median
Head	0.76	0.85	0.79	0.75	0.78	0.76	0.84	0.75	0.78	0.77
Upper Spine	0.71	0.89	0.88	0.88	0.88	0.88	0.93	0.85	0.86	0.88
Lower Spine	0.72	0.43	0.88	0.88	0.88	0.32	0.93	0.85	0.74	0.87
Pelvis	0.74	0.91	0.88	0.88	0.89	0.89	0.92	0.85	0.87	0.88
Upper Arm (L)	0.70	0.71	0.68	0.69	0.72	0.70	0.74	0.70	0.71	0.70
Upper Arm (R)	0.48	0.69	0.73	0.43	0.60	0.44	0.79	0.63	0.60	0.61
Forearm (L)	0.72	0.74	0.72	0.74	0.55	0.73	0.75	0.69	0.70	0.73
Forearm (R)	0.50	0.66	0.70	0.43	0.53	0.42	0.78	0.58	0.58	0.56
Hand (L)	0.40	-0.13	0.51	0.61	0.52	0.21	0.14	0.16	0.30	0.31
Hand (R)	0.49	0.64	0.53	0.50	0.61	0.67	0.48	0.48	0.55	0.52
Thigh (L)	0.69	0.91	0.88	0.88	0.90	0.89	0.92	0.87	0.87	0.89
Thigh (R)	0.74	0.84	0.84	0.80	0.83	0.79	0.89	0.85	0.82	0.84
Shank (L)	0.78	0.89	0.84	0.82	0.84	0.87	0.89	0.85	0.85	0.84
Shank (R)	0.71	0.89	0.87	0.87	0.86	0.88	0.89	0.85	0.85	0.87
Foot (L)	0.73	0.87	0.86	0.82	0.85	0.86	0.88	0.84	0.84	0.86
Foot (R)	0.73	0.90	0.88	0.84	0.86	0.89	0.90	0.85	0.86	0.87
Average	0.66	0.72	0.78	0.74	0.75	0.70	0.79	0.72	0.73	0.75

**Table 3:** Turn-detection of the sensor locations.

Location	Trial 1	Trial 2	Trial 3	Trial 4	Trial 5	Trial 6	Trial 7	Trial 8	Total (Avg.)
Upper Spine	100%	100%	100%	129%	100%	100%	100%	100%	104%
Lower Spine	100%	0%	100%	100%	100%	0%	100%	100%	75%
Pelvis	100%	100%	100%	100%	100%	100%	100%	100%	100%
Thigh (L)	129%	100%	100%	100%	100%	100%	100%	100%	104%
Thigh (R)	200%	129%	114%	100%	171%	114%	286%	171%	161%
Shank (L)	86%	100%	100%	100%	100%	100%	100%	100%	98%
Shank (R)	100%	100%	100%	100%	100%	100%	100%	100%	100%
Foot (L)	100%	100%	100%	100%	100%	100%	100%	100%	100%
Foot (R)	100%	100%	100%	100%	100%	100%	100%	100%	100%

**Table 4:** Summary of performance analysis.

**Turns**	**Turn 1 (R)**		**Turn 3 (R)**		**Turn 5 (R)**	
**Attributes**	**Time Duration (s)**	**Max Pressure Ratio**	**Time Duration (s)**	**Max Pressure Ratio**	**Time Duration (s)**	**Max Pressure Ratio**
Trial 1	2.32	0.89	2.07	0.85	2.69	0.85
Trial 2	2.25	0.94	1.71	0.88	1.86	0.78
Trial 3	2.49	0.80	2.02	0.72	1.94	0.72
Trial 4	2.62	0.93	1.79	0.78	2.14	0.77
Trial 5	2.34	0.93	1.81	0.77	1.74	0.76
Trial 6	2.30	0.92	1.80	0.83	2.05	0.70
Trial 7	2.28	0.83	1.85	0.82	1.74	0.67
Trial 8	2.39	0.89	1.97	0.69	1.75	0.66
**Turns**	**Turn 2 (L)**		**Turn 4 (L)**		**Turn 6 (L)**	
**Attributes**	**Time Duration (s)**	**Max Pressure Ratio**	**Time Duration (s)**	**Max Pressure Ratio**	**Time Duration (s)**	**Max Pressure Ratio**
Trial 1	2.05	0.81	1.62	0.92	3.10	0.97
Trial 2	1.82	0.88	1.57	0.83	1.50	0.89
Trial 3	1.80	0.88	1.62	0.83	1.55	0.87
Trial 4	1.72	0.87	1.57	0.87	1.39	0.89
Trial 5	1.61	0.81	1.52	0.76	1.46	0.87
Trial 6	1.85	0.76	1.39	0.85	1.74	0.84
Trial 7	1.80	0.87	1.51	0.88	1.53	0.88
Trial 8	1.77	0.86	1.42	0.89	1.50	0.90

**Table 5:** Summary of hypothesis tests for the lateral-asymmetric performance analysis.

IMU Sensor		Foot Pressure Sensors	
Null ($H_{0}$)	$\mu_{L}^{*}=\mu_{R}^{*}$	Null ($H_{0}$)	$\mu_{L}^{+}=\mu_{R}^{+}$
Alternative ($H_{1}$)	$\mu_{L}^{*}\neq \mu_{R}^{*}$	Alternative ($H_{1}$)	$\mu_{L}^{+}\neq \mu_{R}^{+}$
Critical points (α=0.05)	2.01	Critical points (α=0.05)	2.03
*t*-statistics	4.27	*t*-statistics	2.62
Results	$H_{0}$ rejected	Results	$H_{0}$ rejected

**Table 6:** Self-assessment score for each trial.

Trial	Trial 1	*	Trial 2	Trial 3	Trial 4	Trial 5	Trial 6	Trial 7	Trial 8
Self-assessment (out of 10)	2.5	3.5	3.5	4.5	5	5	5	5	6
Time duration	13.85	N/A	10.71	11.42	11.23	10.48	11.13	10.71	10.80

Note: N/A = not available.

**Figure A1:**
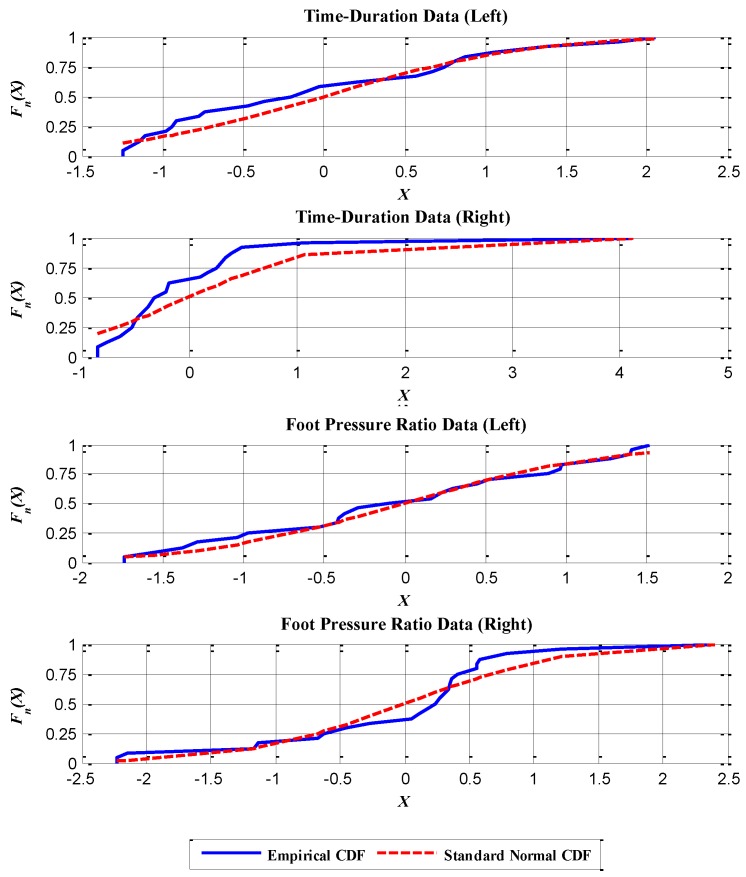
Plot of the empirical cumulative distribution function (cdf) and the standard normal cdf for a visual comparison of the K-S test result.

## Abbreviations

The following abbreviations are used in this manuscript:

IMU: Inertial measurement unit
FIS: FÉDÉRATION INTERNATIONALE DE SKI
3-D: 3-dimensional
IoT: Internet of Things
KNSU: Korea National Sports University
K-S test: Kolmogorov–Smirnov test
CV: Critical value

## Appendix A: Hypothesis Tests for Lateral-Asymmetry Analysis

### A.1. K-S Test

We conducted K-S tests to check the normality of the two data samples: time duration and maximum foot pressure ratio. The K-S test is basically a type of hypothesis test with a null hypothesis that the sample data come from a population with a standard normal distribution. The alternative hypothesis is that the sample data are not from a standard normal distribution. By means of the K-S test, the null hypothesis is rejected if the K-S statistic is larger than the critical value (CV). The critical value in this test is 0.269 for 24 sample data with a significance level *α* = 0.05 [41]. Finally, the results of the K-S tests are listed in Table A1, and Figure A1 is the graphical representation of the results. Note that the K-S statistic is the absolute value of the maximum difference between the empirical cumulative distribution function (cdf) and the hypothesized cdf, such that:

(A1) $$D^{*}=\max_{x}\left(\left|\hat{F}\left(x\right)-G\left(x\right)\right|\right)$$

That is, in Equation (A1), $D^{*}$ is the K-S statistic, $\hat{F}\left(x\right)$ is the empirical cdf and G(x) is the hypothesized cdf, where *x* indicates the data point. The $\hat{F}\left(x\right)$ and G(x) are compared in Figure A1. More details on the K-S can be found in [41,42].

Finally, because the K-S statistics in Table A1 are less than the critical value, the normality of the sample data is now confirmed.

**Table A1:** Summary of the K-S test result.

K-S Test	K-S Statistics	CV (*α* = 0.05)
Time duration (R)	0.146	0.269
Time duration (L)	0.231	0.269
Foot pressure ratio (R)	0.106	0.269
Foot pressure ratio (L)	0.186	0.269

### A.2. Checking the Variance of Population Data

Once we confirmed the normality of the sample data, the next step was to check whether the population variances of two comparing samples are basically the same to conduct a two-sample *t*-test. Consequently, we conducted a hypothesis test with the null hypothesis that the two population variances are basically the same and an alternative that the two population variances are not the same. This test is often called a two-sample *F*-test, and the two samples compared are left and right data in each category, the time duration and the maximum foot pressure ratio. The equations below are the basic form of our hypothesis tests and the test statistics $F_{0}$. $\sigma_{L}^{2}$ and $\sigma_{R}^{2}$ are the population variances of the test attribute of left and right turns, whereas $s_{L}^{2}$ and $s_{R}^{2}$ are the sample variances of the test attribute of left and right turns.

(A2) $$H_{0}:\sigma_{L}^{2}=\sigma_{R}^{2}\;\mathrm{vs}.\;H_{1}:\sigma_{L}^{2}\neq \sigma_{R}^{2}\;\mathrm{and}\;\;F_{0}=\frac{s_{L}^{2}}{s_{R}^{2}}$$

Because there are 24 data points for each sample, with a significance level α=0.05, $F_{0.975}\left(n_{L}-1=23,n_{R}-1=23\right)=0.43$ and $F_{0.025}\left(n_{L}-1=23,n_{R}-1=23\right)=2.31$.

- Hypothesis test for the variances of the time duration data:
  Following Equation (A2), the null hypothesis, $H_{0}$, is that there is no significant difference in the population variances of the time durations between left and right turns ${\sigma_{L}^{*}}^{2}={\sigma_{R}^{*}}^{2}$. The alternative, $H_{1}$, is that there is a significant difference in the population variances of the time durations between left and right turns ${\sigma_{L}^{*}}^{2}\neq {\sigma_{R}^{*}}^{2}$. Finally, the calculated test statistic, $F_{0}^{*}$, is 0.743, that the null hypothesis cannot be rejected because $F_{0.975}\left(23,23\right)=0.43<F_{0}^{*}=0.743<F_{0.025}\left(23,23\right)=2.31$.
  Thus, there is no significant difference in the population variances of the time duration between left and right turns.
- Hypothesis test for the variances of the maximum foot pressure ratio:
  Following Equation (A2), the null hypothesis, $H_{0}$, is that there is no significant difference in the population variance of the maximum foot pressure ratio between left and right turns ${\sigma_{L}^{+}}^{2}={\sigma_{R}^{+}}^{2}$. The alternative, $H_{1}$, is that there is a significant difference in the population variance of the maximum foot pressure ratio between left and right turns (${\sigma_{L}^{+}}^{2}\neq {\sigma_{R}^{+}}^{2}$). Finally, the calculated test statistic, $F_{0}^{+}$, is 3.40 that the null hypothesis is rejected because $F_{0.025}\left(23,23\right)=2.31<F_{0}^{+}=3.40$.
  Thus, there is a significant difference in the population variances of the maximum foot pressure ratio between left and right turns.

In summary, the result of the K-S test shows that the normality of the population data is guaranteed by the sample data of the time duration and the maximum foot pressure ratio. In the hypothesis tests for the variances, the results show no difference in the population variances of the time duration, whereas a significant difference was seen in the population variances of the maximum foot pressure ratio.

## Appendix B: Adaptation Effect of Training

To confirm the correlation between two factors with a comparably small sample size of eight, we conducted a hypothesis test by comparing the *t*-statistic with a *t*-distribution of n−2 degrees of freedom, where *n* indicates the number of samples. The two factors for comparison are the number of trials and the time duration. For the general form of our hypothesis test, the null hypothesis is that there is no correlation between the two factors, and the alternative is that there is a correlation between the two factors. The equations below are our hypothesis test and its test statistics $T^{*}$. $\rho^{*}$ is the true correlation between two factors. $r^{*}$ is the sample correlation value calculated from the data of $n^{*}=8$ samples. For the significance level, we used an *α* value of 0.10 for the two-sided *t*-test.

(B1) $$H_{0}:\rho^{*}=0\;\mathrm{vs}.\;H_{1}:\rho^{*}\neq 0\;\mathrm{and}\;\;T^{*}=\frac{r^{*}\sqrt{n^{*}-2}}{\sqrt{1-{r^{*}}^{2}}}$$

- Hypothesis test for the number of trials *vs.* time duration:
  In the hypothesis test from Equation (B1), the null hypothesis, $H_{0}$, is that there is no significant correlation between the number of trials and the time duration. The alternative, $H_{1}$, is that there is a significant correlation between the number of trials and the time duration.
  Finally, the calculated *t*-statistics is |−2.373|=2.373 with $r^{*}=-0.696$ and the degree of freedom $n^{*}-2=6$; with a significance level (*α*) of 0.10, $T_{0.05}=1.943$. Because $T_{0.05}=1.943<T^{*}=2.373$, the null hypothesis is rejected. By this analysis, the causal relationship between the number of trials and the time duration is confirmed.

## Appendix C: Summary of the Survey

We conducted two surveys, Part 1 before and Part 2 after the experiment. A self-assessment of performance was conducted after each trial.

**Table C1:** Summary of the survey.

**Question 1: Are there any personal characteristics that might affect the performance?**
• It is quite demanding to maintain against the forces acting on the left foot or leg, because of the aftereffects of inveteratedisc surgery on the left lumbar.
• For the best performance, some period of adaptation to the experimental equipment and the course would be required.
• Thorough inspection of the snow surface is mandatory for the best performance (*i.e.*, distribution and quality of snow on the surface).
• Because I could not thoroughly inspect the experimental course, I expected that this would have a negative effect on performance.
**Question 2: Are there any comments for the experiments?**
• Because the experimental course was short and enough time was given for rest after each trial, there was less effect of fatigue when performing the turns during the whole experiment. Rather, I felt that my performances improved as I gradually adapted to the experimental devices and factors, such as snow quality and snow distribution.
• I believe that the performance was greatly affected by the quality of the snow among many other factors that might affect performance.
• In my point of view, there was more snow on the surface than ice, which is not a favorable environment to perform carving turns.
• The pressure sensors in the boots did not influence significantly the performance during the experiments. However, I do not want to use them for daily training.
• The IMU sensors attached to the feet distracted from the control of skis while performing the turns.
**Question 3: Evaluate yourself in a range from one to 10 (10 for the best) for each trial.**
• Please refer to Table 6 in Section 5
